# Carneous Mole

**Published:** 1908-10-17

**Authors:** 


### Obstetrics.

### CARNEOUS MOLE.

The conversion of a human embryo and its coverings into a solid mass, or mole, by hæmorrhage into all its components is by no means a scarce occurrence. Its pathology, however, and often its diagnosis is obscure, whilst its symptoms may be puzzling and misleading. This particular pathological change occurs at a very early period of pregnancy, seldom after eight weeks and often earlier; at the same time, however, it must be admitted that the exact determination of the age of a mole is often impossible, because of the disappearance of the fœtus, whose length would be a guide. The appearance of a carneous mole is usually quite characteristic. It is, as its name implies, a solid mass, usually pink or red on the outside, and conforming somewhat to the shape of the uterine cavity. It has a broad end corresponding with the fundus, and a narrow end corresponding with the internal os. On section it is laminated, and the component layers vary in colour from dark purple black to red or pink. The darkest portion is often the centre, and may be "tarry" in consistence, whilst the outer portions are often quite hard.

Sometimes there is a cavity in the centre lined by a smooth membrane which is in reality the amnion; but as often as not this has broken down and liquefied in the blood which has been effused. Only seldom can the embryo be seen attached by a swollen umbilical cord to the amnion. The term "blighted ovum" was applied by older writers to those specimens in which the embryo could be seen. It is clear, however, that the essential change is the same—namely, hæmorrhage into the coverings of the embryo; and it is only in the slighter cases that the amniotic cavity and embryo persist. The key to the pathology of the affection lies in the microscopic appearances. If a section be made transversely through a carneous mole, it is found to consist of decidua, chorion, with villi and sometimes amnion, converted into a solid mass by blood which has been effused into these structures. The decidua is broken up by the hæmorrhage into islets and masses of decidual cells, with remnants of uterine glands, large areas of blood clot often occurring in a field of the microscope without any decidual cells to be seen. The same may be said of the chorion; villi can be seen surrounded by masses of blood clot, and always show changes referable to their death by deprivation of their natural blood supply. The villi are often swollen, owing to changes in their connective tissue, and the coverings of the villi, especially the syncytium, show necrosis with fragmentation of their nuclei. Masses of nuclear material can be seen here and there, forming shapeless lumps, larger than normal nuclei and evidently due to aggregation of fragmented nuclear material. In spite, however, of these changes, the chorionic villi remain very characteristic structures, and are recognisable as such for long periods of time, long after decidual cells have broken up and lost their distinguishing characters. The strands of fibrin in the blood clot form very fantastic appearances, often giving rise to spurious structures which resemble capillary blood-vessels. The underlying cause of this out-pouring of blood into the embryonic coverings is sometimes obscure, but in general the great determining factor is previous uterine congestion and corporeal endometritis. In this condition the blood-vessels of the mucous membrane are dilated and have exceedingly thin walls, and the result is that very slight changes in the circulation of the uterus are sufficient to cause rupture and effusion of blood. In most cases this blood tears up the chorionic villi and separates the decidua, thus constituting a threatened abortion. In the minority of cases for some reason the blood does not escape, but remains in the substance of the decidua and amongst the villi.

The interesting point is that the formation of a mole is so slow and gradual that uterine contractions are not set up at once. It seems as if the uterus becomes used to the presence in it of a solid mass, and no particular irritation is set up for a time. This state of things, however, cannot last for many weeks, and sooner or later the mole plays the part of a foreign body, sets up uterine contractions, and is eventually expelled. This, however, may not occur for a month or perhaps two, rarely longer than this. It is in those cases in which the mole is retained that interesting points arise as to diagnosis. The usual history is that, after missing one or two periods and believing herself to be pregnant, the patient notices that the signs of pregnancy are not progressive. The abdomen does not enlarge, the breasts, which were enlarging, cease to do so and become flabby, and although there is no specific complaint the patient does not feel well. If an examination is made at this time the uterus will be found to be smaller than the period of amenorrhœa would seem to suggest. The uterus at the same time is harder than a normal pregnant uterus would be, and does not show the typical softening of the lower segment. If there is a discharge it may be rather watery and sometimes blood-stained. The diagnosis is often impossible, and sometimes it may not even be possible to say whether a normal progressing pregnancy is present. As a rule a waiting policy has to be adopted, unless some serious symptom demands exploration of the uterus. The symptom which may demand it is infection of the mole, with consequent decomposition and absorption of toxic products. As a rule, however, before this happens uterine contractions are set up and the mole is spontaneously expelled. If this happy result does not occur and the contents of the uterus are obviously septic, the only useful treatment is dilatation of the cervix and removal of the mole under an anæsthetic. If this operation is refused, one or more laminaria tents may be introduced, in the hope of starting uterine contractions and consequent expulsion of the contents of the organ.

